# Chemical entrapment and killing of insects by bacteria

**DOI:** 10.1038/s41467-020-18462-0

**Published:** 2020-09-14

**Authors:** Louis K. Ho, Martin Daniel-Ivad, Swathi P. Jeedigunta, Jing Li, Konstantin G. Iliadi, Gabrielle L. Boulianne, Thomas R. Hurd, Craig A. Smibert, Justin R. Nodwell

**Affiliations:** 1 Department of Biochemistry, University of Toronto, 661 University Avenue, Toronto, ON M5G 1M1 Canada; 2 Department of Molecular Genetics, University of Toronto, 661 University Avenue, Toronto, ON M5G 1M1 Canada; 3 The Hospital for Sick Children, Peter Gilgan Centre for Research and Learning, 686 Bay St., Toronto, ON M5G 0A4 Canada

**Keywords:** Biochemistry, Chemical biology

## Abstract

*Actinobacteria* produce antibacterial and antifungal specialized metabolites. Many insects harbour actinobacteria on their bodies or in their nests and use these metabolites for protection. However, some actinobacteria produce metabolites that are toxic to insects and the evolutionary relevance of this toxicity is unknown. Here we explore chemical interactions between streptomycetes and the fruit fly *Drosophila melanogaster*. We find that many streptomycetes produce specialized metabolites that have potent larvicidal effects against the fly; larvae that ingest spores of these species die. The mechanism of toxicity is specific to the bacterium’s chemical arsenal: cosmomycin D producing bacteria induce a cell death-like response in the larval digestive tract; avermectin producing bacteria induce paralysis. Furthermore, low concentrations of volatile terpenes like 2-methylisoborneol that are produced by streptomycetes attract fruit flies such that they preferentially deposit their eggs on contaminated food sources. The resulting larvae are killed during growth and development. The phenomenon of volatile-mediated attraction and specialized metabolite toxicity suggests that some streptomycetes pose an evolutionary risk to insects in nature.

## Abstract (web summary)

*Streptomyces* bacteria produce metabolites with diverse biological activities. Here, Ho *et al*. show that some of these metabolites are volatile terpenes that attract fruit flies, while other metabolites can kill the insect’s larvae upon ingestion of bacterial spores.

## Introduction

The actinomycetes are a diverse phylum of Gram-positive bacteria found all over the earth. They have been isolated from most terrestrial habitats including soils and microbiomes on destinations as diverse as the Lechuguilla cave network^1^, the mantles of sponge-eating sea slugs^2^, the rhizosphere and beyond^3–9^.

A defining feature of these organisms is their specialized metabolism. This ensemble of biochemical pathways generates thousands of small molecules that have biological activity against other organisms. Many of these compounds have antibacterial or antifungal activity and are used clinically as antibiotics^10–12^. Others, such as the DNA-intercalating agents daunorubicin, actinomycin and cosmomycin, can serve as a defence mechanism against bacteriophage infection^13^. A smaller number of these compounds are active against eukaryotic cells and some of these are used as anticancer drugs, immune suppressants and anthelminthic drugs^14^.

Many actinomycetes have intimate contact with insects and other multicellular organisms^15^. For example, they have been identified in the microbiomes of fruit flies and other Diptera, honeybees, diamondback moths and silkworms^16–19^. This is perhaps not surprising as insects and streptomycetes are abundant, have co-existed for more than 479 million years, and are certain to have played important roles in each other’s evolutionary history^20^. Indeed, most streptomycetes produce geosmin, a volatile compound that can serve as a chemical attractant for springtails and mosquitoes^21,22^. The production of volatile terpenes is conserved in distantly related bacterial phyla including the *Myxococcus* and some fungi, so it is likely that such interactions are common in nature^23,24^.

Actinomycetes frequently provide chemical advantages to their insect hosts. For example, *Acromyrmex octospinosus* (leaf-cutter ants) depend on a fungus called *Leucogaricus* as a food source. This fungus however is susceptible to a microfungal weed called *Escovopsis*. A mutualistic relationship of the leaf-cutters with candicidin- and antimycin-producing streptomycetes selectively inhibits *Escovopsis* thereby protecting the ant’s food source^25^. A similar relationship exists between *Dentroctonus frontalis* (bark beetles) and an actinobacterial strain that produces mycangimicin, which also inhibits the growth of undesirable fungal species^26^. Another example is the solitary wasp genus *Philanthus* which secretes *Streptomyces philanthi* from glands in their antennae. In this instance, the insects cover their larval broods with the bacteria to protect them from infection by other microbes during their early development^27^. All the interactions between actinomycetes and insects that have been characterized to date are beneficial to the insect.

The beneficial nature of these interactions is striking given that, in addition to the antibacterial and antifungal compounds, many streptomycetes make compounds that are toxic to insects. This includes DNA-intercalating compounds such as daunorubicin and actinomycin D, which were discovered through their action against *Saccharomyces cerevisiae*^28^. These molecules are toxic to most living organisms and are used as front-line chemotherapy drugs. Indeed, milbemycin^29^, avermectin^30^, prasinon^31^, doramectin^32^, nanchangmycin^33^ and spinosads^34^ are selectively toxic to invertebrates, including insects. At the most extreme, the compounds milbemycin, avermectin and the spinosads specifically target biochemical pathways that are unique to invertebrates. It seems a priori that such compounds would confer significant risk to the choice of actinomycetes species as mutualistic partners for insects. To our knowledge, this question, and the role of these toxic compounds in nature more generally, has not been previously addressed.

In this work, we used laboratory strains of the fruit fly *Drosophila melanogaster*, as well as outbred lines and more distantly related fruit fly species, as a model system to explore the interactions between insects and the actinomycetes of the genus *Streptomyces*. We demonstrate that many *Streptomyces* species produce metabolites that are toxic to the fly. Importantly, we find that the spores of *Streptomyces* species that produce these toxic molecules kill larvae that ingest them and that the mechanisms of killing are specific to the toxic metabolites they produce. Finally, we demonstrate that under laboratory conditions, the volatile compounds produced by streptomycetes, can attract flies to feed on food sources that are contaminated by toxic spores. The result of this attraction is that they lay their eggs there with the ensuing death of their progeny. This demonstrates that a widely distributed actinomycetes species can have such a deleterious effect on insects.

## Results

### Several streptomycetes make insecticidal metabolites

To compare the prevalence of specialized metabolites that act on prokaryotes, unicellular eukaryotes and multicellular eukaryotes we created small molecule extracts from 56 *Streptomyces* strains and tested them for inhibitory activity against *Escherichia coli, Bacillus subtilis, Saccharomyces cerevisiae, Candida albicans* and *Drosophila melanogaster*. To avoid bias based on known specialized metabolites in the well-established model systems we selected streptomycetes randomly from the Wright Actinomycetes Collection^35^. All the strains we used were wild isolates that had not been previously characterized—at the outset of this work the genome sequences and specialized metabolic potential of all of them were unknown.

**Fig. 1:**
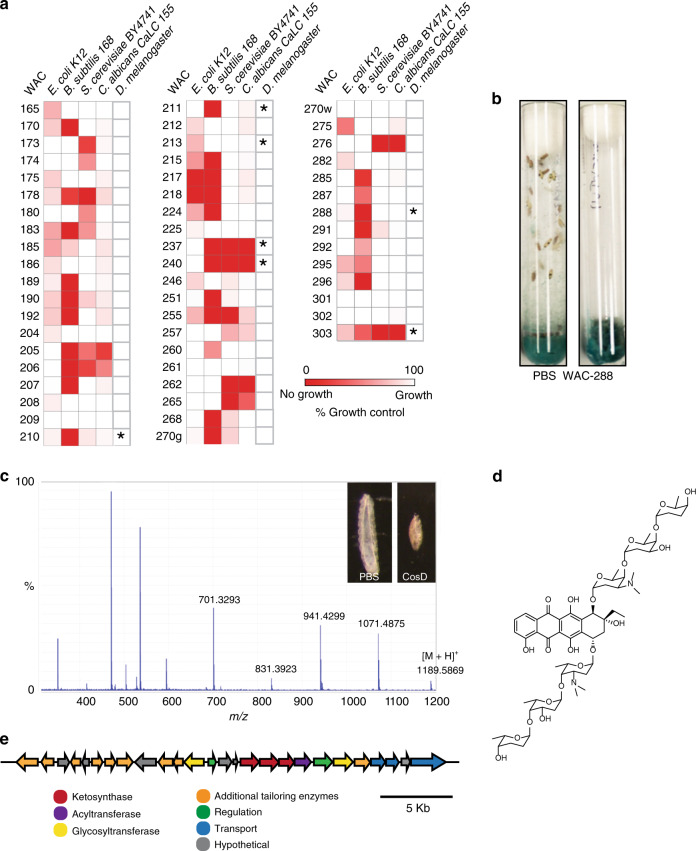
Identifying insecticidal bioactivity in actinomycete extracts. **a** A set of 56 crude extracts derived from the Wright Actinomycete Collection (WAC) were generated and tested for growth inhibition against model prokaryote (*E. coli, B. subtilis*), yeast (*S. cerevisiae*, *C. albicans*) and insect (*D. melanogaster*) model organisms. Visualized is each extract’s inhibitory activity against corresponding model organisms where values of microbial growth are relative to the DMSO control. Asterisks (*) indicate extracts that had larvicidal activity whereby no larvae developed into adult flies. **b** Shown is the activity of an extract from WAC-288 which inhibited the development of *D. melanogaster* larvae into adult flies. **c** The molecule with larvicidal activity was purified from WAC-288 and its identity was confirmed via tandem MS/MS fragmentation analysis. The parent ion and the masses of corresponding fragments were identified (Supplementary Fig. 2). **d** Structure of the antibiotic cosmomycin D. **e** Biosynthetic gene cluster identified from the sequenced genome of WAC-288 corresponding to cosmomycin D production.

Focusing first on the microbial screens (Fig. 1a) we assessed the ability of extracts to inhibit >90% growth and found that 25 extracts were active against *B. subtilis*, two were active against *E. coli*, 8 were active against *S. cerevisiae* and 5 were active against *C. albicans*. This is consistent with many previous screens^36^ and confirms that antibacterial activity against Gram-positive bacteria is very common while activity against eukaryotic microbes and Gram-negative bacteria is less so.

To investigate the bioactivity of the extracts against *Drosophila*, we seeded twenty newly hatched first instar larvae into tubes containing a control food source or a food source supplemented with *Streptomyces* extract. We monitored their progression through to pupation and eclosion to adult flies for 14 days. Extracts from the 7 strains WAC-240, -237, -303, -288, -210, -211 and -213 had potent inhibitory activity against the growth and development of *D. melanogaster* such that no larvae developed into adult flies. In their place, we observed dead, desiccated larvae with arrested growth at various stages of development. One potent insecticidal extract was derived from strain WAC-288 (Fig. 1b). This work demonstrates that insect-toxic specialized metabolites are relatively common; they are similar in number to those that are active against Gram-negative bacteria and lower fungi.

### Toxicity of *Streptomyces* spores

**Fig. 2:**
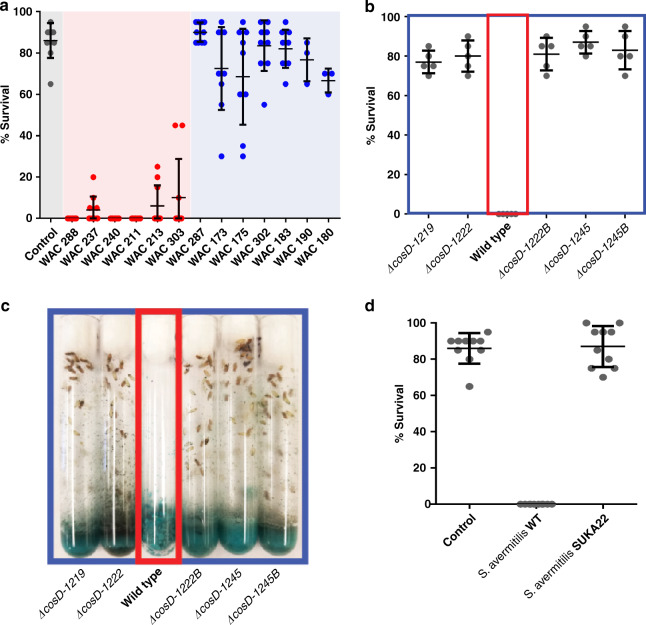
Actinomycetes pose a threat to larval viability due to the production of insecticidal metabolites. **a** Survival rates of *D. melanogaster* larvae that were fed live spores of various actinomycete strains. The effect of WAC strains that produced fly-toxic extracts are in red compared to strains that did not produce fly-toxic extracts in blue (*n* = 10). **b**, **c** Survival of larvae that were fed wild-type WAC-288 compared to strains with different cosmomycin D biosynthetic genes deleted (*n* = 5). **d** Survival of larvae that were fed the avermectin producer *S. avermitilis* wild type compared to the effect of the avermectin deficient mutant SUKA22 (*n* = 10). The measure of centre for error bars indicate the mean of each data set. Error bars of all graphs indicate the standard deviation of all biological replicates.

We then asked whether spores of streptomycetes that produce insect-toxic extracts are themselves harmful to *Drosophila*. We chose 6 *Streptomyces* strains that had generated fly-toxic extracts (WAC-211, -213, -237, -240, -288 and -303) and another 6 (WAC-173, -175, -183, -190, -287 and -302) that generated non-toxic extracts and conducted feeding experiments with fly larvae. We prepared spores from these 12 strains, washed them twice in PBS to remove media and then added each of them to fly growth medium. We seeded the spore-treated and control tubes with larvae and allowed them to feed and develop for 14 days. After a suitable period we observed that spores of strains that did not make toxic extracts (WAC-173, -175, -183, -190, -287 and -302) had little or no effect on fly development: 60–100% of the larvae in each culture proceeded through normal development to generate healthy adults after 14 days. In contrast, spores of strains that generated toxic extracts (WAC-211, -213, -237, -240, -288 and -303) had lethal effects on the larvae such that all of them had arrested development and died prior to developing to adulthood (Fig. 2a). WAC-288 spores were particularly toxic to larvae. We observed reduced mobility of the spore-fed larvae within 3–6 h of ingestion and a rapidly worsening condition such that all larvae were motionless, desiccated and non-viable within ~24 h.

To determine whether this effect could be observed in fruit flies other than domesticated Canton-S *D. melanogaster* strains, we repeated the spore-feeding experiment with 6 outbred fruit fly strains from the *D. melanogaster* Genetic Reference Panel (DGRP)^37^. As shown in Supplementary Fig. 1, the result was the same: the six outbred *D. melanogaster* strains were equally sensitive to WAC-288 spores such that all larvae were killed prior to completing development. We further repeated the experiment with *D. virilis, D. suzukii, D. yakuba, D. simulans* and *D. pseudoobscura*. Again the result was the same: the larvae of all five species died in the presence of the *Streptomyces* spores; none of them completed development to adults.

### Cosmomycin D is the causative agent of killing by WAC-288

To understand the basis of this toxicity we isolated the insecticidal compound from WAC-288 using bioactivity-guided fractionation. The compound absorbed light at *λ* = 494 nm, a characteristic of anthracycline molecules^38^. It had a parent ion mass-to-charge ratio of *m/z* 1189.5869 [M + H]^+^. We carried out tandem mass spectrometry on this compound and observed four other key fragments *m/z* 1071, 941, 831 and 701 (Fig. 1c, Supplementary Fig. 2). This mass fragmentation pattern has been reported previously for the red-pigmented compound cosmomycin D^13,39–42^. As a complementary approach, we sequenced the WAC-288 genome and analyzed the 7.4 Mbp sequence using AntiSMASH that predicts specialized metabolite biosynthetic gene clusters. We found that the strain is predicted to encode 24 biosynthetic gene clusters for specialized metabolites (Supplementary Table 1). One of these biosynthetic gene clusters is predicted to generate cosmomycin D (Fig. 1d, e). This is based on a high degree of sequence homology in all encoded proteins (97%) as well as a similar gene organization to the known cosmomycin D biosynthetic gene cluster in *S. olindensis*^43^ (Supplementary Fig. 3).

To determine whether the cosmomycin D biosynthetic genes were responsible for the toxicity of WAC-288 spores to fly larvae we constructed mutations in *orf1219* (encoding a PadR-like regulator), *orf1222* (encoding a β-keto acyl synthase) and *orf1245* (encoding a predicted cluster-situated regulator) (Supplementary Fig. 4, Supplementary Table 2). We confirmed that the three mutants were defective in producing cosmomycin D by LC-MS (Supplementary Fig. 5) and compared the capacity of their spores to kill fly larvae to the parent strain. The result (Fig. 2b, c) indicated that all three mutants had lost their ability to kill larvae. The yields of viable adult flies were identical to the negative control in all three cases with 70%–95% mature flies. In contrast, spores from the wild-type parent, WAC-288, killed all the larvae in the culture. This confirms that cosmomycin D is responsible for the insecticidal activity of WAC-288 spores.

To determine whether this phenomenon occurs with other well-characterized streptomycetes we compared it to the effect of spores of *S. avermitilis*, the producer of avermectin, an inhibitor of invertebrate locomotion^44,45^. As a control, we used SUKA22, an *S. avermitilis* mutant that is unable to produce this compound^46^ (Fig. 2d). Consistent with the mode of action of avermectin, *S. avermitilis* spores were also toxic to the fly larvae. The phenotypic effect, however, was distinct from that of WAC-288. In this case, we observed complete paralysis of all larvae within 10 min of ingestion: the larvae ceased locomotor movement, a well-known effect of avermectin. Consistent with the cause of this being avermectin, SUKA22 had no effect on larval phenotype, survival or development. Taken together, these data demonstrate that the spores of streptomycetes that produce insecticidal compounds are toxic to invertebrates and confirms that this toxicity is due to specific specialized metabolites.

### Mechanism of spore-fed lethality is determined by toxic metabolites

Cosmomycin D is a DNA intercalator and many of these molecules cause cell death in eukaryotes. This, for example, is why doxorubicin, a molecule that shares the same anthracycline scaffold as cosmomycin D is used as a chemotherapeutic drug^47^. In mammalian cells, DNA damage leads to the activation of the apoptosis initiator caspase-9 and executioners: caspase-7 and caspase-3. This process also occurs in *Drosophila melanogaster* however it is carried out by Dronc, a capsase-9 homolog, and two caspase-3 homologs Dcp-1 and DrICE^48^.

**Fig. 3:**
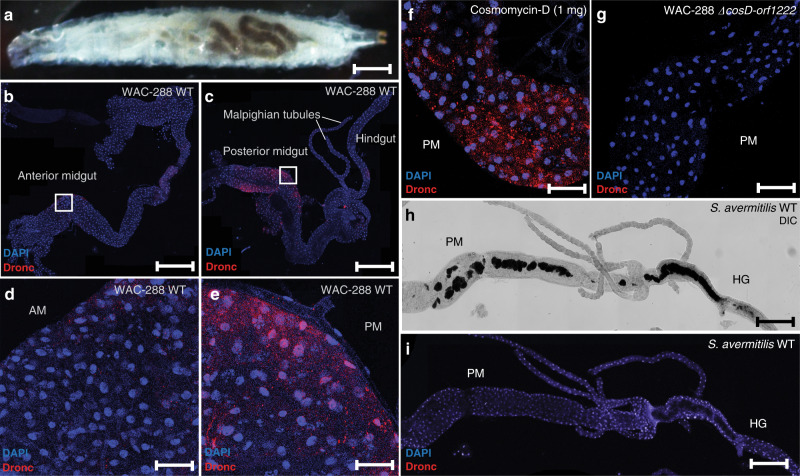
Cell death in *D. melanogaster* is triggered by the consumption of cosmomycin D producing spores. **a** Third instar larvae that have fed on media containing spores of WAC-288. After feeding, larvae were dissected and processed for fluorescence microscopy for the detection of activated Dronc which is the caspase-9 homolog in *Drosophila* (Scale bar = 1 mm) Specific regions of the (**b**) anterior and (**c**) posterior/hindgut were identified and visualized (Scale bars = 1 mm). Images at a higher magnification of the (**d**) anterior midgut shows lower levels of Dronc activation compared to the (**e**) posterior midgut which displays higher levels of activation (Scale bars = 50 µm). **f** Activation of Dronc in posterior midgut of larvae that fed on food containing 1 mg of pure cosmomycin D isolated from WAC-288 (Scale bar = 1 mm). **g** No activation in the posterior midgut of larvae that were fed spores of the cosmomycin D deficient mutant *∆cosD-orf1222* (Scale bar = 1 mm). **h** Spores of *S. avermitilis* are visible and accumulate in the posterior midgut and hindgut (Scale bar = 1 mm). **i** Lack of Dronc activation within the indicated regions of larval guts that were fed *S. avermitilis*. Each result was individually reproduced three times with similar results (Scale bar = 1 mm).

To determine whether WAC-288 spores induce the activation of caspase proteins we carried out an experiment in which we fed spores of the cosmomycin D producing parent or the cosmomycin D defective *ΔcosD-orf1222* mutants to third instar larvae. We dissected out the digestive tracts of the larvae 6 h after feeding and them and stained them with the antibody #9661, which binds to the activated caspase-9 homolog Dronc in *Drosophila melanogaster*^49^. The results demonstrated that feeding spores of WAC-288 resulted in the activation of Dronc primarily in cells of the posterior end of the midgut and hindgut, suggestive of a cell death-like phenomenon in this region of the larvae’s digestive tract (Fig. 3a–e). We observed the same phenomenon when we fed pure cosmomycin D to larvae (Fig. 3f). In contrast, feeding with the *ΔcosD-orf1222* mutant spores did not result in Dronc activation in cells of the digestive tract (Fig. 3g). This suggests that WAC-288 kills fly larvae by compromising the cells of their midgut and hindgut digestive tracts.

To determine whether this effect is shared by *S. avermitilis* we carried out the same experiment comparing the effect of wild-type *S. avermitilis* and WAC-288. We again found that a phenotypic effect of feeding with *S. avermitilis* was much quicker than with WAC-288—the larvae were paralyzed within minutes. In marked contrast to the effect of WAC-288, dissected digestive tracts from larvae that had fed on wild-type *S. avermitilis* for the same duration did not display the cell death-like activity (Fig. 3h, i). This is consistent with the distinct mechanism of action of avermectin, which is muscle paralysis via the inhibition of the invertebrate-specific glutamate-gated chloride channel at neuromuscular junctions^50^. These data demonstrate that the mechanisms of invertebrate killing by *Streptomyces* spores are determined by specialized metabolites produced by each species.

### Attraction to volatile compounds leads flies to a toxic food source

**Fig. 4:**
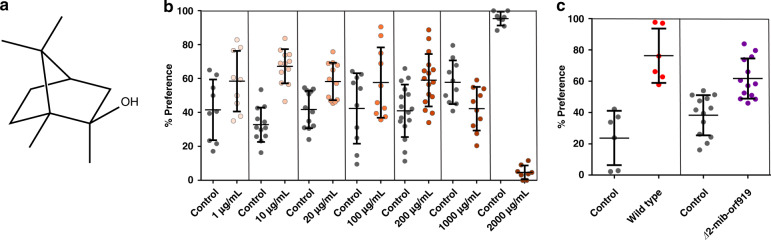
Adult flies are attracted to *Streptomyces* cultures and 2-MIB. **a** Structure of 2-MIB. **b** Preference of adult flies to pure 2-MIB. Darker colours indicate a higher concentration of 2-MIB. The number of flies that preferred positions closer towards either the control or 2-MIB odorant was calculated as a percentage of total flies within the T-maze. **c** Adult flies placed in an enclosed space selected between a control (PBS) or contaminated food source. Contaminated food sources contained liquid culture of either the wild type or a 2-MIB deletion mutant of WAC-288. Solid horizontal lines represent the measure of centre as the mean of each data set. Error bars indicate the standard deviation of all biological and technical replicates. A two-tailed *P*-value of 0.014 (*n* = 6) (wild-type) and 0.123 (*n* = 13) (2-MIB) was calculated where a *p* < 0.05 is considered significant.

WAC-288 produces the volatile compound 2-methylisoborneol (2-MIB) (Fig. 4a). Indeed, the production of 2-MIB is widespread and highly conserved throughout the actinobacterial phyla (Supplementary Fig. 6). While the unrelated volatile terpene geosmin is known to influence insect behaviour via repulsion^51^ and attraction^21,22^, the biological effect of 2-MIB on insects has only recently been described^21^. We, therefore, tested pure 2-MIB for effects on adult flies in a T-maze and found that high concentrations (2 ×  10^3^ µg/mL at the source) repelled them (4.7% preferred 2-MIB, *p* < 0.001) (Fig. 4b, Supplementary Movie 1) whereas low concentrations (10 µg/mL at the source) resulted in a subtle attraction towards the compound (67% of total flies preferred 2-MIB over the control, *p* < 0.001)

We then carried out an experiment to determine whether flies were attracted or repelled by spore-contaminated food sources and whether this was due to the production of 2-MIB. Flies were placed in a closed container with two food sources: one source was a control that lacked *Streptomyces* spores, the other contained either wild-type WAC-288 or a mutant unable to produce 2-MIB. Flies could choose between the different food sources for 24 h with their choice being evident by their final physical location at the experimental end point as well as the location where they had laid their eggs. We also observed the subsequent success of their progeny in both conditions by clearing the adult flies and allowing the eggs to hatch and undergo development. We observed more flies near the medium containing wild-type WAC-288 spores compared to medium lacking added bacteria (76% wild type, 24% control, *p* < 0.05). This was consistent with the attractive properties of 2-MIB at lower concentrations of the pure molecule (10 µg/mL) and by recent reports by Becher et al.^21^. In contrast, in a similar experiment in which the food contained the 2-MIB defective strain, this was attraction was greatly reduced or abolished (55% ∆*2-mib-orf919*, 44% control, *p*-value: not significant) (Fig. 4c). In a sense, therefore, the adults were ‘trapped’ in the tube containing the medium contaminated with the wild-type strain as a result of their attraction to the volatile terpene. The consequence of the attraction to the wild-type strain was significant. Any eggs that were laid in the uncontaminated medium grew normally and generated adult progeny, as expected. In contrast, all the progeny that were deposited and hatched in the presence of WAC-288 died prior to pupation (Supplementary Fig. 7). In this case, therefore, the consequence of attraction was a complete failure to generate viable progeny.

To determine whether this attractive effect is widespread in the *Drosophila* genus we repeated this food choice experiment with *D. melanogaster* outbred lines as well as five other distantly related *Drosophila* species (Supplementary Fig. 8, Supplementary Note 1). As with the domesticated laboratory strain of *D. melanogaster*, the outbred strain (DGRP cross 1), *D. virilus, D. yakuba, D. simulans* and *D pseudoobscura* were attracted towards the WAC-288 contaminated food source. The one exception was *D. suzukii* which exhibited a slight but not statistically significant repulsion from the WAC-288 contaminated food source. Therefore, most outbred and domesticated species of *Drosophila* are attracted to 2-MIB producing streptomycetes including those that produce compounds that are exceptionally harmful to them and their larvae.

## Discussion

We find that Actinobacterial insecticidal metabolites are relatively common. They are less common than anti-Gram-positive antibacterials but they are as common as antibiotics against Gram-negative bacteria and lower eukaryotes such as *S. cerevisiae* and *C. albicans*. We demonstrate a clear correlation between the production of these molecules in cell-free extracts and the capacity of ingested spores to kill fly larvae. The observed lethality is due to the presence of the biosynthetic genes for the insecticidal compounds. Indeed, the onset of toxicity and mechanism of action between two strains WAC-288 and *S. avermitilis* is determined by the metabolites in question: cell death in the digestive tract over a 24-h period and rapid muscle paralysis within minutes respectively. We also find that *Streptomyces* volatile compounds can serve to attract adult flies such that they preferentially lay their eggs on a contaminated food source with catastrophic results for their progeny. Notably, both the killing and, attraction effects are conserved in outbred strains of *D. melanogaster* and other *Drosophila* species.

These are unique insights in *Streptomyces-*insect biology. Aside from plant pathogens such as *Streptomyces scabies*^52,53^, and strains such as *S. somaliensis* and *S. sudanensis* which cause extremely rare subcutaneous infections in humans^54,55^, streptomycetes are generally regarded as harmless. They have no genes that are homologous to known virulence determinants or pathogenicity islands^56–58^. Indeed, the existing literature on interactions between streptomycetes and insects indicates that they are either benign substituents of their microbiomes^16^ or, in the case of many insects, highly beneficial mutualists^25–27^.

Our work suggests a new view in which some streptomycetes have the capacity to chemically attract insects and poison them through a diversity of specialized metabolite-mediated mechanisms. In this work, we focused on two molecules that facilitate this effect, however, there are likely many others including well-known molecules like rapamycin which inhibits the TOR pathway^59^, inhibition of transport between the nucleus and cytoplasm by leptomycin B^60^, inhibition of translation by cycloheximide^61^ and many others. None of these relationships is related to that of classical pathogens. One plausible hypothesis is that they are analogous to the relationship between humans and *Clostridia* that cause food poisoning (*Clostridium perfringens*) or frequently fatal infections of the digestive tract (*Clostridium difficile*)^62,63^. The difference being that while *Clostridia* exert their effect via protein-based endotoxins, *Streptomyces* use small molecule-based strategies. At present it is not possible to predict how frequently insects are harmed by streptomycetes in nature, however, we note that several well-characterized strains such as *S. bingchenggensis*, *Saccharopolyspora spinosa* and *S. cineroruber*, all have biosynthetic genes for both 2-MIB and an insecticidal compound. (Table 1). This might suggest that these streptomycetes benefit from attracting and metabolising insects in nature. Indeed, *Streptomyces* are known to be able to metabolize N-acetylglucosamine which is not only a monomer of bacterial cell walls but also a monomer of chitin which is the major constituent of insect biomass^64^.

**Table 1:** Streptomycetes that produce 2-MIB and other compounds that are toxic to insects.

Identified 2-MIB producer	Produced toxin(s)
*Streptomyces bingchenggensis BCW-1*	Meilingmycin, nanchangimicin
*Saccharopolyspora spinosa NRRL 18395*	Spinosad
*Streptomyces griseus subsp. griseus NBRC 13350*	Nonactin, Cycloheximide
*Streptomyces cinereoruber strain ATCC 19740*	Cosmomycin-D

This work raises important questions concerning the relationships between insects and actinobacteria in nature. We wonder whether some eukaryotes harbour cognate resistance genes for widespread actinobacterial toxins. It further suggests that the acquisition of beneficial bacterial species by ants, bark beetles, wasps and other insects must be fraught with risk. The attractive effect of 2-MIB produced by potentially helpful species would be selective for the insect as it would help it to find a microbe that protects its food source or its offspring. An alternative hypothesis is that this attractive effect could be selective for the streptomycete as it would provide it with an opportunity to use the insect’s carcass as a food source. There may be unknown mechanisms that allow insects to differentiate between beneficial and toxic streptomyces species. Alternatively, and we suspect that this is more likely, it may be that most or all insect-streptomycetes relationships are established via direct transmission from parent to offspring. Regardless, we suggest that the high frequency of insect toxicity, along with a strongly attractive odorant, is likely to have had a significant evolutionary impact on both insects and streptomycetes.

## Methods

### Extract preparation

Fifty-six strains from the Wright Actinomycete Collection (WAC) were selected for screening. Strains were cultured on 25 mL petri dishes of Maltose-Yeast Extract-Malt Extract (MYM) agar medium^65^ for seven days. Following growth, lawns were macerated, submerged in *n-*butanol and sonicated for 10 min. Extracts were left overnight then the solvent was filtered and fully evaporated. Concentrated crude extracts were suspended in 500 µL DMSO (for microbes) or dH_2_O (for flies) for screening in 96-well plates containing bacteria or yeast in either LB or YPD liquid broth respectively. Three technical replicates were carried out in the antimicrobial and fly assays. Extract hits were identified as a 75% reduction in the average OD_600_ of growth compared to the DMSO control. Morpheus https://software.broadinstitute.org/morpheus (Broad Institute) was used to generate the heat map in Fig. 1a

### Screening against *D. melanogaster*

*Drosophila melanogaster* has been used previously to investigate human pathogens^66–68^—we have therefore used it as a model system to explore interactions between insects and streptomycetes. Stocks of *D. melanogaster* Canton-S (Provided by Craig Smibert at the University of Toronto) were maintained and used for all assays unless otherwise indicated. Embryos were collected on apple juice agar supplemented with nipagin and yeast paste. Embryos were synchronized by collecting within a 6-hour time-window after being deposited. Once embryos had hatched, the assay was prepared by placing 20 first instar larvae into Instant *Drosophila* Medium (Carolina Biological Supply Company Formula 4-24) mixed with equal volumes of suspended extract-containing solution in 0.75 mL dH2O in 12 × 75 mm (5 mL, round bottom) polystyrene tubes with cotton plugs. Larvae were incubated for 14 days on a 12-h day-night cycle in a 60% humidity-controlled room at 25 °C. For initial screens, assays were carried out using three technical replicates of extracts. Hits were considered as tubes containing no adult flies after 14 days.

### Isolation, purification and elucidation of cosmomycin D

A 5 µL aliquot of WAC-288 spore stock was sub-cultured on MYM agar for 7 days. Colonies were selected and grown in 4 × 700 mL liquid R5 media (−50% maltose)^69^ shaking at 200 rpm in baffled flasks for 7 days. Cultures were sonicated for 10 min and centrifuged at 20,000 × *g* to remove the cell pellet. Spent media was filtered and continuously mixed with 20 g of Diaion® HP-20 Resin overnight. Resin was packed into an empty column cartridge and flash chromatography was performed using the Reverleris X2 system (Grace). Reverse-phase HPLC was carried out with a 250 ×4.6 mm C18 5 μm column (Phenomenex) on the Alliance HPLC system (Waters). LC-MS/MS data were obtained using an Acquity UPLC (Waters) with an inline/Xevo G2-S qTOF (Waters) and processed with MassLynx V4.1 and MZmine 2 Software. Please refer to supplementary methods for complete cosmomycin isolation details. Small-scale larval assays to detect bioactivity were carried out in a microcentrifuge cap with 15 μL dH_2_O and 5 μL suspended aliquots of flash and HPLC fractions. Twenty-first instar larvae were placed in the droplet and caps were covered in parafilm for 2 days.

### Spore feeding

Spore-feeding assays were carried out similarly as described with extract screening. Each strain was grown on 25 mL of MYM agar and tested individually as ten biological replicates. After growth, spores were suspended in a 0.85% saline solution, centrifuged 13,000 × *g* for 5 min, and resuspended in PBS buffer prior to addition to fly media. Survival was quantified as a percentage of the number of adults that eclosed after 14 days. There may have been some growth of the *Streptomyces* in the fly medium during these experiments however if so, it was not robust enough to observe aerial hyphae or pigmented spore layers that are readily visible during growth on plates (Supplementary Fig. 9).

### Inactivation of cosmomycin D and 2-MIB biosynthesis

Three genes of the cosmomycin D biosynthetic gene cluster (*cosD*-*orf1219*, -*orf1222*, -*orf1245*) and one biosynthetic gene within the 2-MIB cluster (*2-mib-919*) were selected for disruption. They were individually introduced into separate pOJ260 plasmids which has an Apr^R^ cassette^70,71^. Plasmids were transformed into *E. coli* ET12567 and conjugated into WAC-288. Apr^R^ strains were selected and correct deletions were confirmed with PCR. Please refer to supplemental methods for more details.

### Microscopy and antibody staining

To visualize spores in the larval gut, third instar larvae were washed in PBS for 20 min. They were then placed in spore-containing fly food and allowed to feed for 6 h. Larvae were washed in PBS; whole guts were dissected. For fluorescence microscopy, guts from spore-fed larvae were dissected in ice-cold PBS, fixed in 4% paraformaldehyde in PBST (PBS, 1% v/v triton) for 45 min, rinsed with PBST, incubated in PBST for 1 h, incubated in PBSTB (0.2% triton, 1% BSA) for 1 h and incubated with agitation overnight at 4 °C in PBSTB containing antibodies against cleaved (activated) caspase-3 (Asp175) #9661 (Cell Signaling Technology #9661) at a 1:400 dilution. Guts were removed from the primary antibody solution and washed twice for 30 min in PBSTB, incubated with PBSTB and Cy™3 AffiniPure Donkey Anti-Mouse IgG (Jackson ImmunoResearch, Code: 715-165-151) diluted 1:500 overnight at 4 °C. Guts were washed with PBST three times for 30 min each. DAPI (NucBlue™ Fixed Cell ReadyProbes™ Reagent) was used as a counterstain during the last wash then washed three additional times for 10 min in PBS. Buffer was removed and samples were suspended in Vectashield (Vector Laboratories, H-1000) mounting medium for 1 h at room temperature or stored at 4 °C. Samples were visualized on a Lecia confocal fluorescent microscope and images were minimally processed in Fiji (ImageJ 1.52p). A single plane was acquired for all images approximately in the middle of the tissue. Tile scan images were taken of guts at 10X using the 405 and 552 laser lines. The same laser power was used to acquire all images for consistency. Assays were reproduced in duplicate.

### Preference assays

A T-maze apparatus^72^ was used for testing pure 2-MIB (Toronto Research Chemicals) dissolved in mineral oil. Each of the 10–14 trials were sequentially alternated on both sides in darkness at room temperature under infrared light for two minutes. Two-tailed *p*-values were generated based on preference values which were calculated by dividing the number of flies on each side by the total of flies within the maze. For culture preference assays adult flies were starved for 4 h prior to being placed in a polystyrene 10 cm (height), 10 cm (radius) cylinder enclosure with a mesh net top for 24 h. Flies had to select between food source traps with 1.5 g of fly media with 3 mL of PBS control or the indicated strain grown in liquid R5 culture for 10 days. After a 24-h period, the % preference was calculated by dividing the number of flies in a tube containing the test condition by the total number of flies found in the test and PBS control condition. PBS vs WT preference comprised of six replicates. The result was so significant that more replicates were not warranted. PBS vs. 2-MIB mutant preference was carried out in a total of 13 replicates. The variability made multiple replicates necessary. Two-tailed *p*-values were calculated based on a *T*-test of %Total Preference values of each paired condition. A *p*-value less than 0.05 was considered significant for this study. Tubes were cleared of adult flies and incubated for 14 days to determine progeny survival. Microsoft Excel for Office 365 and GraphPad PRISM 6 was used for all data processing.

### Bioinformatics

The complete genome of WAC-288 was sequenced with PacBio single-molecule real-time sequencing (SMRT) (Genome Quebec). To predict potential biosynthetic gene clusters corresponding to cosmomycin D and 2-MIB production in WAC-288, AntiSMASH^73^ was used. 2-MIB biosynthetic gene clusters were identified in other strains by using BLAST for the 2-MIB terpene synthase gene. Identifying strains that encode both 2-MIB and a possible insect toxin was carried out by querying combined terpene synthase of 2-MIB and core genes encoding compounds that would potentially be broadly toxic to insects^14^. The genomes of the top 50 hits from this query were analyzed via AntiSMASH to detect compounds encoded in the genome with known insecticidal activity or other toxic effects. The genome sequence was deposited to National Center for Biotechnology Information (NCBI) database which is available via the GenBank accession CP027022.1

### Reporting summary

Further information on research design is available in the Nature Research Reporting Summary linked to this article.

## Supplementary information

Supplementary Information

Peer Review File

Description of Additional Supplementary Files

Supplementary Movie 1

Reporting summary

## Data Availability

The genome sequence that supports this study has been deposited in GenBank with the accession code CP027022.1. The authors declare that all other data supporting the findings of this study are available within the paper and its supplementary files within the source data. Source data are provided with this paper.

## Source data

Source Data
