# Supplementary material for: Chemical entrapment and killing of insects by bacteria
Source: Nat Commun. 2020 Sep 14;11:4608. doi: 10.1038/s41467-020-18462-0 (PMC7490686; doi:10.1038/s41467-020-18462-0)
Supplement: Supplementary file 1 — Supplementary Information [file 41467_2020_18462_MOESM1_ESM.pdf]

Supplementary Information for

Chemical entrapment and killing of insects by bacteria.

Louis K. Ho[1], Martin Daniel-Ivad[1], Swathi Jeedigunta[2], Jing Li[1], Konstantin G. Iliadi[3], Gabrielle L. Boulianne[2,3], Thomas Hurd[2], Craig A. Smibert[1,2], and Justin R, Nodwell[1,*]

This .PDF file includes:

Supplementary Figures 1 to 9

Supplementary Tables 1 to 3

Supplementary Note 1

Supplementary Methods

Supplementary References

1. Department of Biochemistry, University of Toronto, 661 University Avenue, Toronto, Ontario, Canada M5G 1M1
2. Department of Molecular Genetics, University of Toronto, 661 University Avenue, Toronto, Ontario, Canada M5G 1M1
3. The Hospital for Sick Children, PGCRL, 686 Bay St., Toronto, Ontario, Canada M5G 0A4

*To whom correspondence should be addressed.
Email: justin.nodwell@utoronto.ca

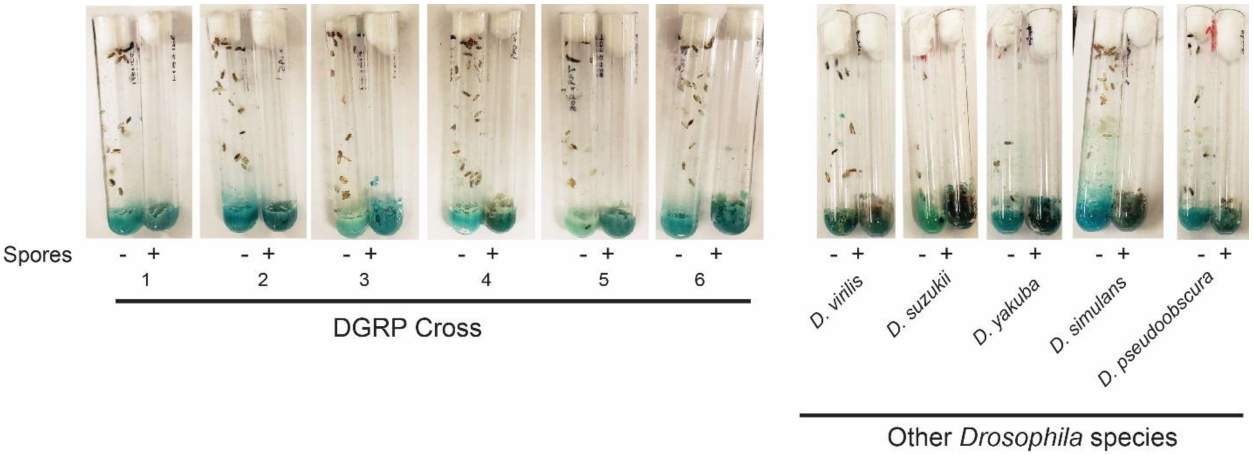

Supplementary Fig. 1 *D. melanogaster* outbred lines as well as distantly related *Drosophila* species are susceptible to *Streptomyces* spores. Larvae of various *Drosophila* species were placed in and fed on either control fly media containing PBS (-) or WAC-288 spore-containing fly media (+). End points were taken after 14 days of incubation where differences in the number of larvae developing into adult flies was visible. Feeding *Drosophila* species with WAC288 spores in each case lead to the entire larvae population having no survival. Each result was individually reproduced three times with similar results.

A

Compound	Composition	Theoretical Mass (Da)	Observed Mass (Da)	Error (mDa)
Cosmomycin D	$[M+H]^+$	1189.5907	1189.5869	3.8
1	M-f4-f2-f7	1071.4801	1071.4875	7.4
2	M-f10-f8	941.4283	941.4299	1.6
3	M-f9-f8	831.3915	831.3923	0.8
4	M-f9-f6	701.3285	701.3293	0.8

B

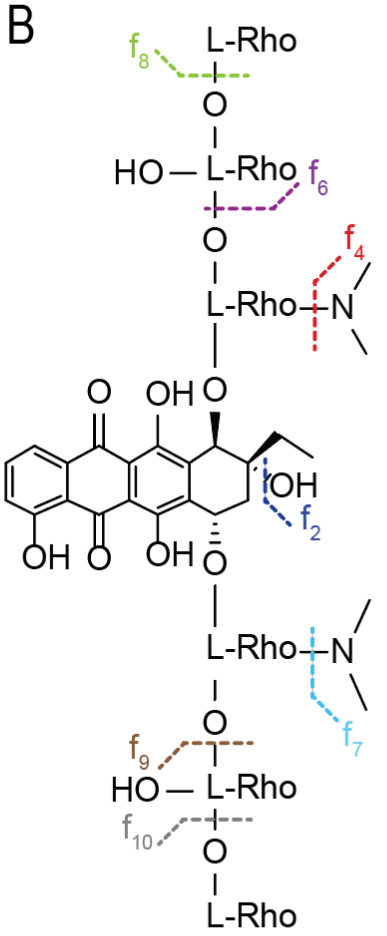

Supplementary Fig. 2 Isolating and elucidating cosmomycin D from WAC-288. (A) With tandem MS/MS, pure cosmomycin D was fragmented yielding several daughter ions in the mass spectrum. Theoretical and observed mass differences between these assignments were negligible. **(B)** Key fragments corresponding to the structure of cosmomycin D were assigned both on the structure as well as indicated on the spectrum.

Supplementary Table 1 Biosynthetic gene clusters found in WAC-288. Summary of the antiSMASH prediction results for the genome of WAC-288. Clusters marked in red indicate compounds of interest in this study.

Cluster	Type	% Similarity	Predicted Molecule
1	Terpene	-	-
2	NRPS	-	-
3	Melanin	-	-
4	NRPS	-	-
**5**	**Terpene**	**100**	**2-methylisoborneol**
6	Terpene	69	Hopene
7	Butyrolactone	-	-
**8**	**Type II PKS-Oligosaccharide**	**97**	**Cosmomycin-D**
9	Butyrolactone-NRPS	-	-
10	Terpene	100	Isorenieratene
11	Bacteriocin	-	-
12	Siderophore	-	-
13	Melanin	-	-
14	Phenazine-NRPS	-	-
15	Type I PKS-NRPS	-	-
16	Siderophore	100	Desferrioxamine B
17	Type I PKS	50	Abyssomicin
18	Type II PKS-Terpene	83	(Spore pigment)
19	NRPS	80	Scabichelin
20	NRPS	-	-
21	Terpene	100	Geosmin
22	Ectoine	100	Ectoine
23	NRPS	9	Laspartomycin
24	Terpene	-	-

A

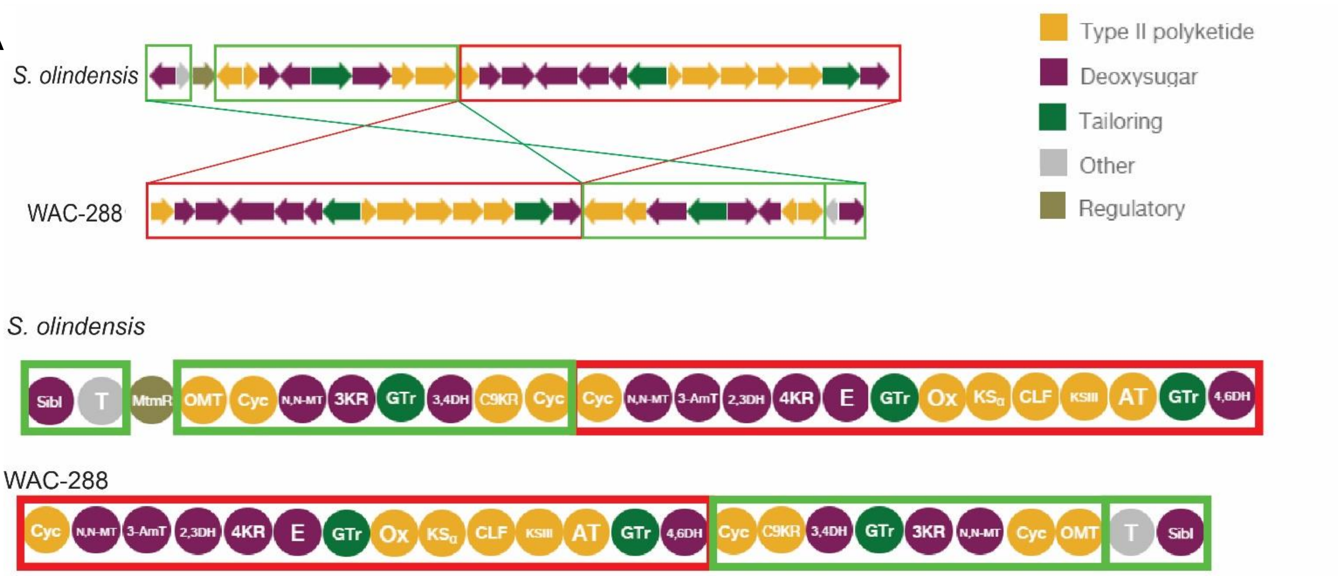

B

Abbreviation	Domain
2,3DH	2,3-dehydratase
3,4DH	3,4-dehydratase
3-AmT	3-aminotransferase
3KR	3-ketoreductase
4,6DH	4,6-dehydratase
4KR	4-ketoreductase
AT	Acyltransferase
C9KR	C9 ketoreductase
CLF	Chain length factor
Cyc	Type II polyketide cyclase
E	Epimerase
GTr (x3)	Glycosyltransferase
KSIII	Ketosynthase III
KSα	Ketosynthase α
N,N-MT	N,N-dimethyltransferase
OMT	Carboxy-O-methyltransferase
Ox	Anthrone-type oxygenase
Ox	Oxidoreductase
Sibl	Glucose-1-phosphate thymidylyltransferase
T	Thiolation

Supplementary Fig. 3 Analyzing the cosmomycin D biosynthetic gene cluster. (A) Comparison of the cosmomycin D biosynthetic gene cluster between *S. olindensis* and WAC-288. The green and red boxes represent two major sections of the gene cluster that have been inverted between the two species. **(B)** Reference table describing PRISM[2] annotated domains for the cluster. Purple represents tailoring enzymes for saccharide chains, yellow boxes are core biosynthetic genes within the Type II polyketide synthase, green are glucosyltransferase domains and grey are other functional domains.

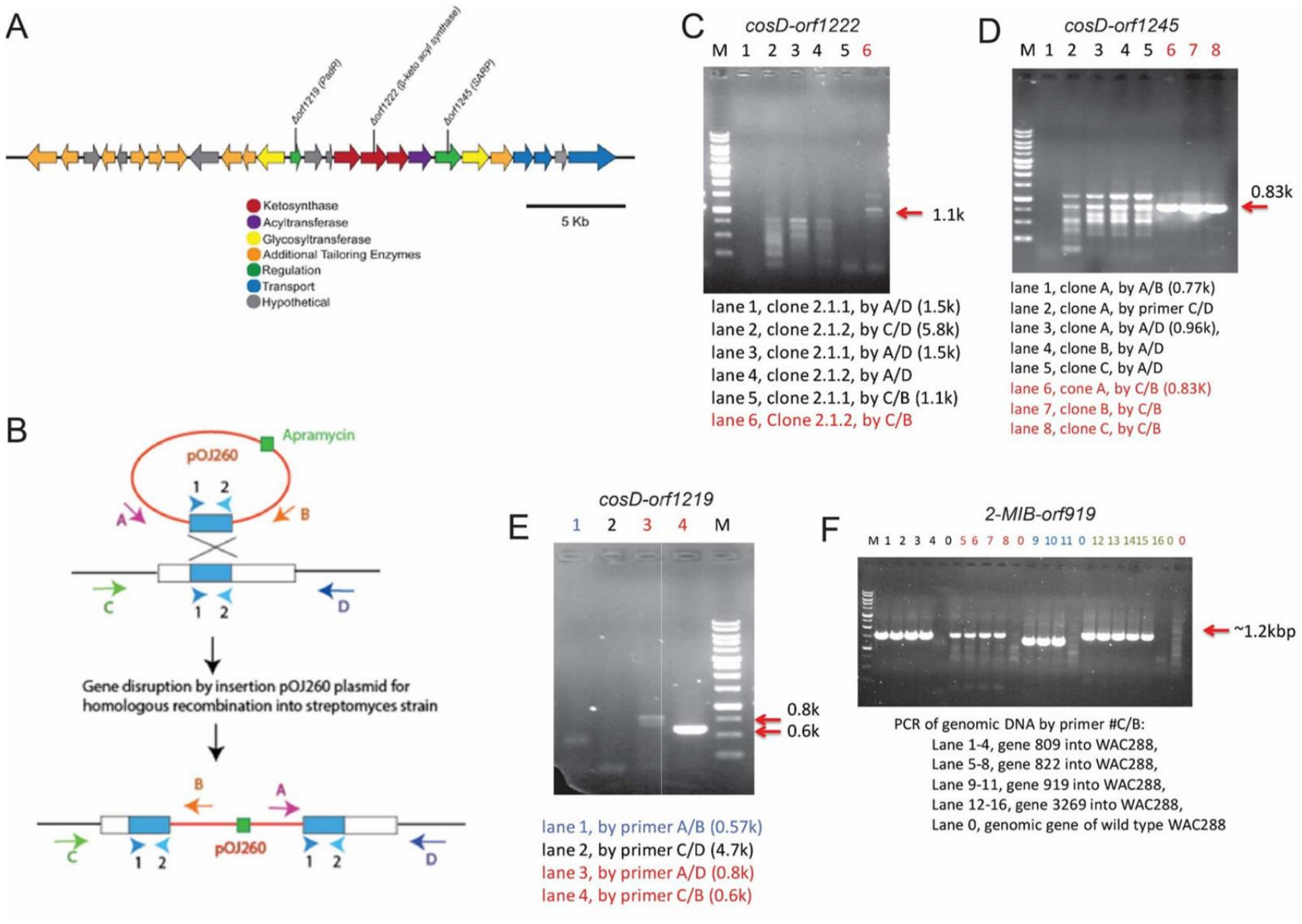
A
Δorf1219 (PadR)
Δorf1222 (β-keto acyl synthase)
Δorf1245 (SARP)
Ketosynthase
Acyltransferase
Glycosyltransferase
Additional Tailoring Enzymes
Regulation
Transport
Hypothetical
5 Kb
B
Apramycin
pOJ260
1 2
A
B
C
D
Gene disruption by insertion pOJ260 plasmid for homologous recombination into streptomyces strain
C
cosD-orf1222
M 1 2 3 4 5 6
1.1k
lane 1, clone 2.1.1, by A/D (1.5k)
lane 2, clone 2.1.2, by C/D (5.8k)
lane 3, clone 2.1.1, by A/D (1.5k)
lane 4, clone 2.1.2, by A/D
lane 5, clone 2.1.1, by C/B (1.1k)
lane 6, Clone 2.1.2, by C/B
D
cosD-orf1245
M 1 2 3 4 5 6 7 8
0.83k
lane 1, clone A, by A/B (0.77k)
lane 2, clone A, by primer C/D
lane 3, clone A, by A/D (0.96k),
lane 4, clone B, by A/D
lane 5, clone C, by A/D
lane 6, cone A, by C/B (0.83K)
lane 7, clone B, by C/B
lane 8, clone C, by C/B
E
cosD-orf1219
1 2 3 4 M
0.8k
0.6k
lane 1, by primer A/B (0.57k)
lane 2, by primer C/D (4.7k)
lane 3, by primer A/D (0.8k)
lane 4, by primer C/B (0.6k)
F
2-MIB-orf919
M 1 2 3 4 0 5 6 7 8 0 9 10 11 0 12 13 1415 16 0 0
~1.2kbp
PCR of genomic DNA by primer #C/B:
Lane 1-4, gene 809 into WAC288,
Lane 5-8, gene 822 into WAC288,
Lane 9-11, gene 919 into WAC288,
Lane 12-16, gene 3269 into WAC288,
Lane 0, genomic gene of wild type WAC288

Supplementary Fig. 4 Deleting Cosmomycin-D and 2-MIB Biosynthesis

Schematic of **(A)** genes for **(B)** insertional fragment mutagenesis. Plasmid pOJ260 with an apramycin resistance gene (Apr^R) was cloned with **(C - E)** cosmomycin D biosynthetic genes or **(F)** 2-MIB terpenes synthase (teal). Target-directed integration of the plasmid by homologous recombination into the chromosomal DNA of WAC-288 inactivated the target gene to effectively abolish cosmomycin D or 2-MIB production. Forward and reverse insertion primers are indicated as blue arrows as 1, and 2 respectively. Primers used to confirm proper insertion are indicated as arrows A-D (See Table 3 below for primer sequences).

Supplementary Table 2 Primers used for gene disruption (1: forward, 2: reverse) and confirmation. Primers used for confirmation (Primers A and B are standard primers of pOJ260[3], C: forward, D: reverse).

Product	ORF	Insert primers	Confirmation Primers
**Cosmomycin D**	1219	1:GGCCAGTGCCAAGCTTCCACACGATTGCTGGTCCTCG	C:GGTACCTGCCTCATGGAGAA
		2:GCGCGGCCGCGGATCCAGGAGATCCGCTCCCTCAGCAG	D:CTCCAGGAAGCCCTCGTC
	1222	1:GGCCAGTGCCAAGCTTGCATCGGGACCAAGCAGTTCTG	C:CGACCGGTACGTCAACATC
		2:GCGCGGCCGCGGATCCCGGTCATGTGGTAGGCGTTGC	D:GTAGCGGTCGGGGTCGAAGG
	1245	1:GGCCAGTGCCAAGCTTGGGAGCTATGGGACGAGGACC	C:TGTCTGAAGCTGACCTCTGG
		2:GCGCGGCCGCGGATCCGAGGTGGTTCACCAGGTTGAC	D:GACGCCGGTGCGGTTCGTG
**2-MIB**	919	1:CGGCCAGTGCCAAGCTTGCTGTAGCGGTAGGTGTTGG	C:GTGGTGGTCGTGTCGGTGC
		2:CGCGCGGCCGCGGATCCCGAAAGCGTTCCCGAAGTC	D:GCACCAGCCCGTTCAGCAC

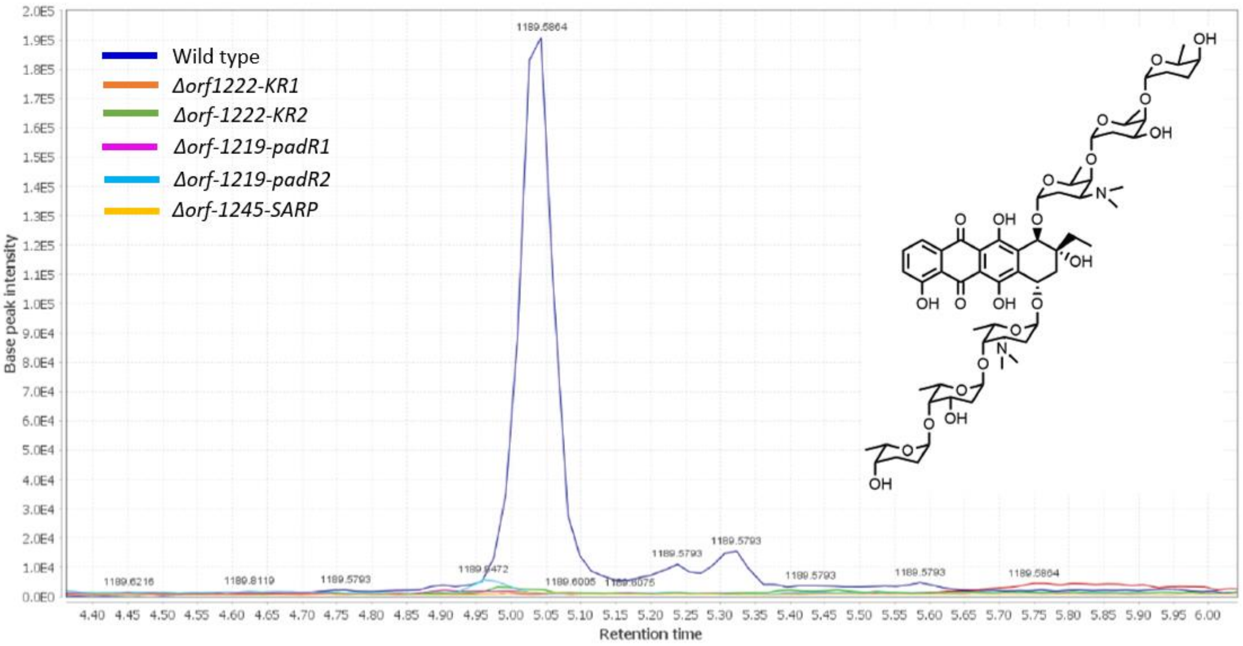

Supplementary Fig. 5 Mutants of WAC-288 are defective in cosmomycin D production. Shown is an ion extracted mass chromatogram corresponding to cosmomycin (m/z 1189.5 +/- 0.5 Da) of crude extracts prepared from each cosmomycin D mutant generated in WAC-288. A peak corresponding to cosmomycin D was only observed in the wild type strain. Each mutant strain was individually repeated twice with similar results.

Supplementary Fig. 6 Species-level phylogenetic analysis of WAC-288

A phylogeny of twelve *Streptomyces* strains was constructed using six housekeeping genes (16S rRNA, *rpoB, atpD, gyrB, recA* and *trpB*). Asterisks (*) indicate the two strains tested in this study: WAC00288 and *S. avermitilis* with experimentally confirmed insecticidal activity via. cosmomycin D and avermectin respectively. This constructed phylogeny suggests that the experimentally confirmed insecticidal activity of spores is not likely to be conserved due to the evolutionary distance between the two the *Streptomyces* species.

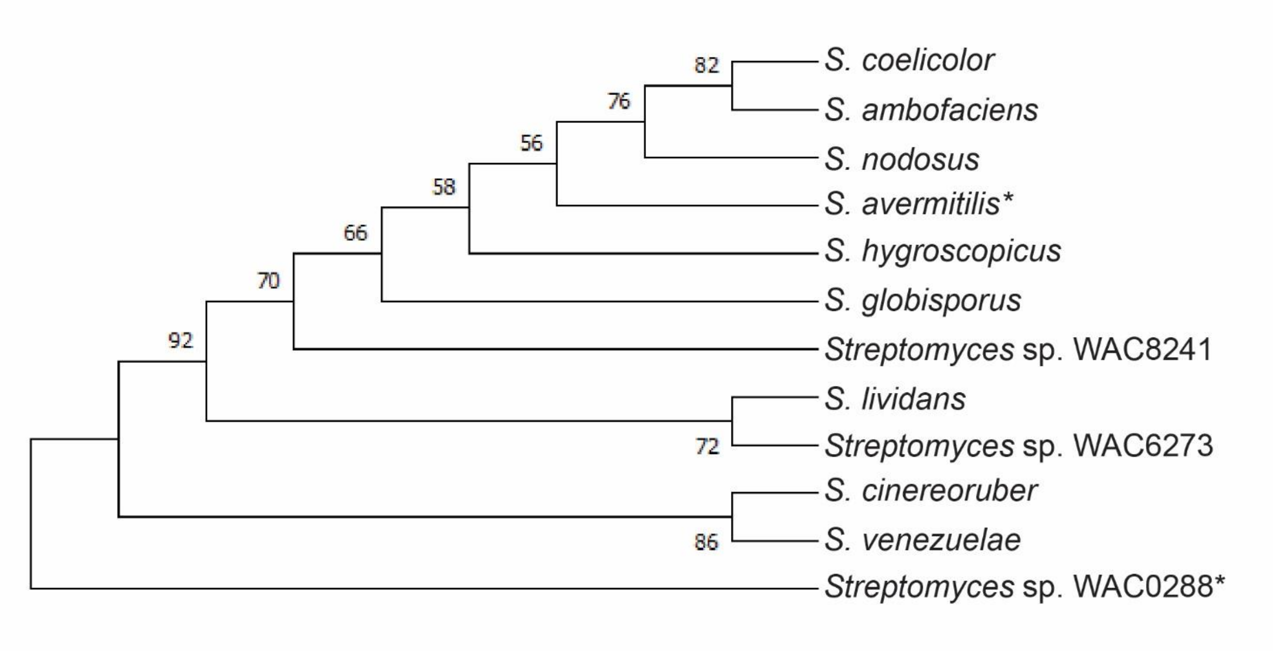

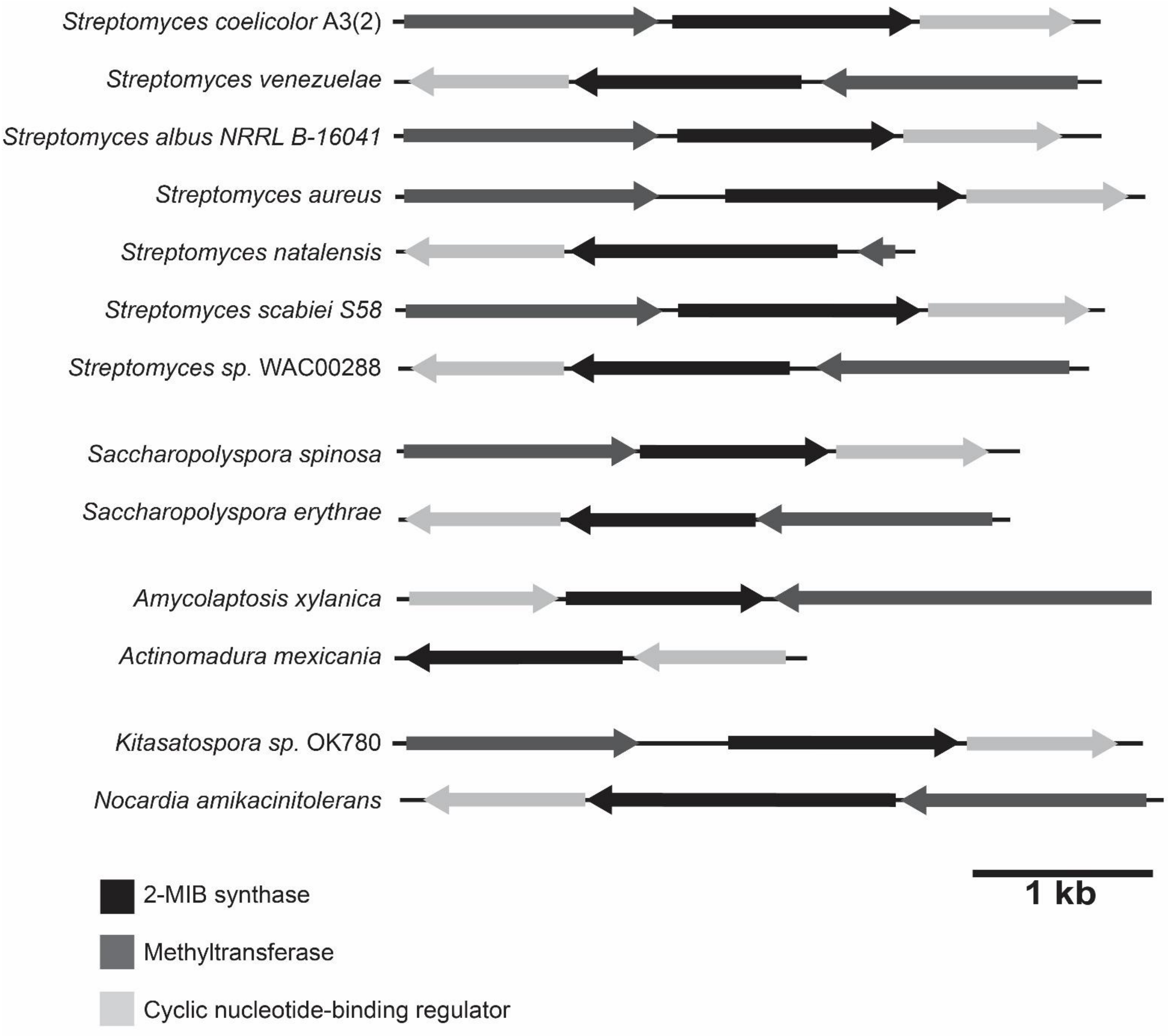

Supplementary Fig. 6 2-MIB production is common amongst actinomycetes. A BLAST search was carried out using the terpene cyclase of 2-MIB in WAC-288. The three-gene biosynthetic cluster of each 2-MIB producer was compared between six well-known *Streptomyces* strains and 6 other actinomycete genera.

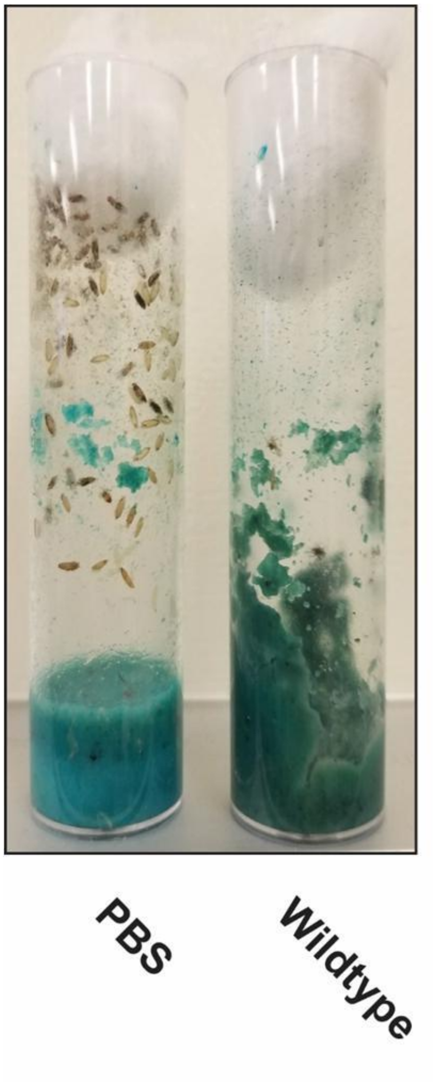

Supplementary Fig. 7 Embryos deposited in contaminated food sources have reduced survival. Following a 24h selection period between a control (PBS) food source and one treated with wild type WAC-288 spores, adult flies were removed from tubes and incubated for an additional 12 days to test whether viable progeny could be generated. Shown are progeny that survived in respective food sources after incubation. Despite more flies having a preference for the contaminated food source, no progeny were able to survive under WAC-288 contaminated food conditions.

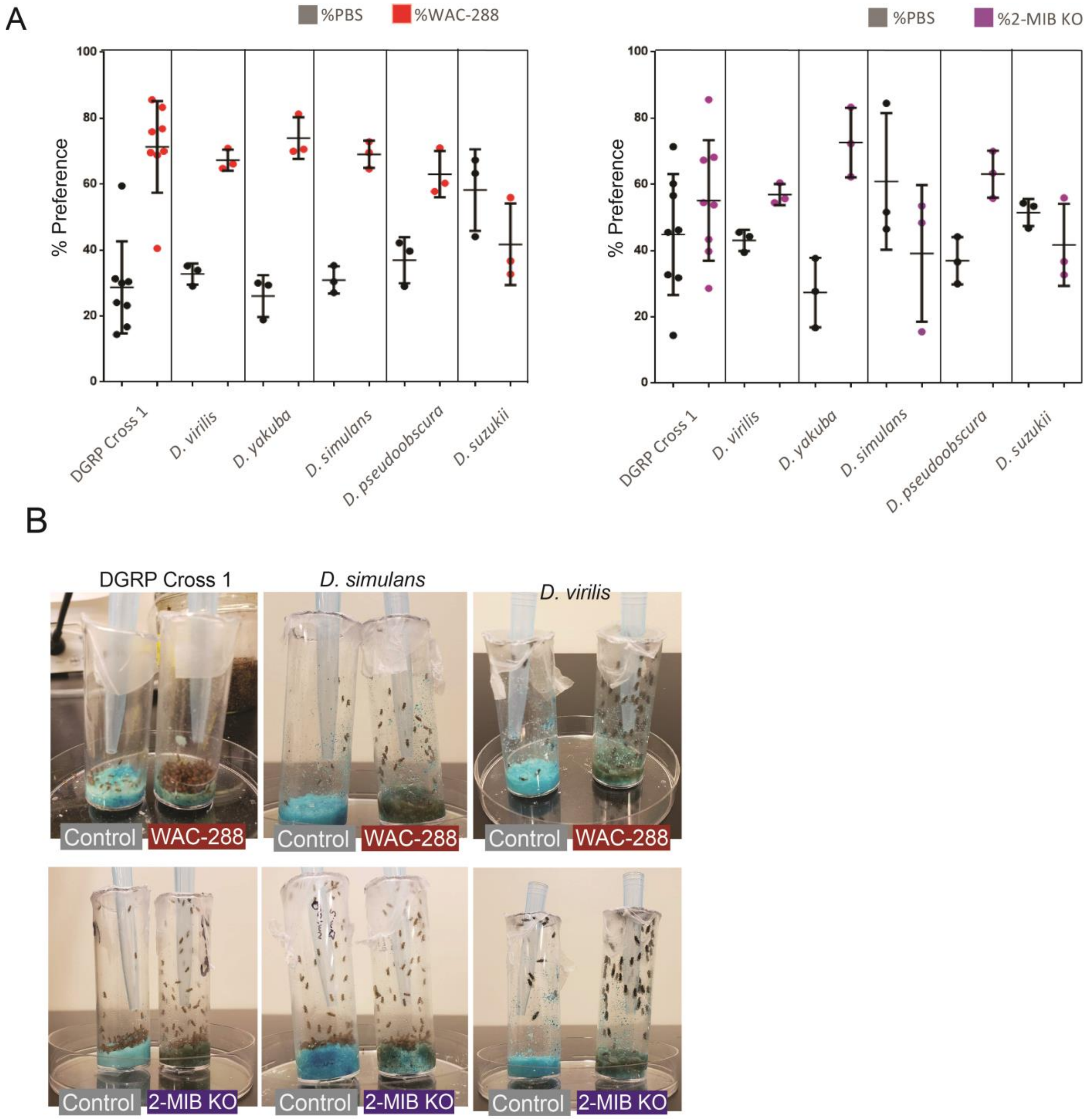
A
%PBS
%WAC-288
% Preference
100
80
60
40
20
0
DGRP Cross 1
D. virilis
D. yakuba
D. simulans
D. pseudoobscura
D. suzukii
%PBS
%2-MIB KO
% Preference
100
80
60
40
20
0
DGRP Cross 1
D. virilis
D. yakuba
D. simulans
D. pseudoobscura
D. suzukii
B
DGRP Cross 1
D. simulans
D. virilis
Control
WAC-288
Control
WAC-288
Control
WAC-288
Control
2-MIB KO
Control
2-MIB KO
Control
2-MIB KO

Supplementary Fig. 8 Several *Drosophila* species are attracted to *Streptomyces* cultures. (A) A variety of *Drosophila* species were trapped over 24 hours a control food source or a wild type WAC-288 spore containing food source to determine fly preference. Food preference is represented by the bar graphs indicating the number of flies found in either the PBS control (grey) or treated (red) food sources. (Right) The same experiment was carried out between a control food source (grey) or food source containing a mutant spores unable to produce 2-MIB (purple). Error bars indicate the standard deviation between at least three biological replicates for each trial. The solid horizontal lines indicate the measure of the mean **(B)** Shown are six representative endpoints of the preference assays that were carried out.

Supplementary Note 1 Preference is conserved in *Drosophila*. We found that 5/6 other species (*D. virilis, D. yakuba, D. simulans, D. pseudoobscura*, DGRP Cross 1 but not *D. suzukii*) were also significantly attracted to food contaminated with a liquid culture of wild type WAC-288. Furthermore, of these flies that were attracted to the wild type culture of WAC-288, three of them lost this preference when given the choice between a control food source and a food source containing the 2-MIB knockout strain. These include the non-domesticated flies (Cross 1), *D. virilis* and *D. simulans*. While not all *Drosophila* species responded similarly—this may be due to their inherent variations in olfaction, it remains clear that the detection of volatile compounds produced by *Streptomyces* is highly conserved in the *Drosophila* genus.

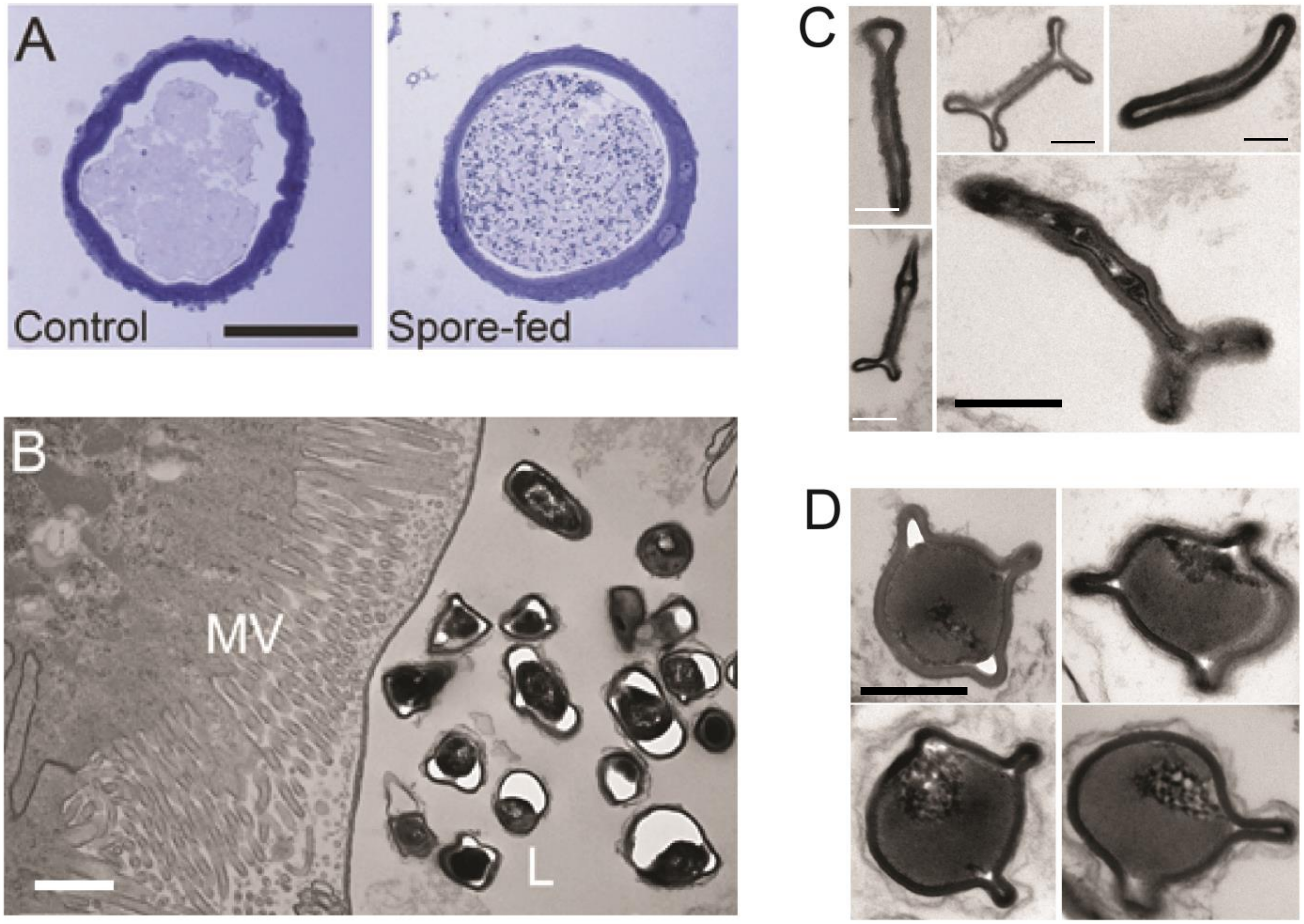

Supplementary Fig. 9 Visualizing spores that ingested by fruit fly larvae. Shown are midgut cross sectional images of dissected fly guts that contain WAC-288 spores. **(A)** (Left) Larvae exposed to food with no spores/PBS control and (right) guts of larvae that fed on spores. Samples were stained with toluidine blue. Scale bar = 50 µm **(B)** Transmission electron micrograph of spore-fed guts. MV: Microvilli, L: Lumen. scale bar = 1 µm **(C)** Hyphae were present in some areas of the lumen (All scale bars = 1 µm) as well as **(D)** possible spores with germ tubes (scale bar = 1 µm).

Supplementary Methods

Purification of cosmomycin D

1. WAC-288 subculture was inoculated into 4L of R5 media
2. Shake 200 rpm at 30ºC for 10 days
3. Cultures were centrifuged at 20,000 x g, supernatant (spent media) filtered and subject to 20g/L Diaion® HSP20 resin, stirring overnight
4. Resin was filtered and packed into a 20g empty flash column cartridge
5. Flash chromatography purification method: Flow rate: 10 mL/min. Spent media adsorbed onto Diaion® HP-20 resin overnight was then packed into an empty 20g column and run through flash chromatography system. MeOH elution was collected between 50 – 90 min:

Time (min)	%H_2O	%MeOH
0	100	0
60	0	100
90	0	100

6. After subsequent evaporation, samples were run through XSelect CSH C18, 5 µm, 4.6 × 150 mm column on an HPLC system (Waters). Cosmomycin-D was collected between 15.77 - 16.36 min:

Time (min)	%H2O	%MeCN
0	90	10
1	90	10
12	80	20
20	70	30
25	60	40
35	5	95
40	90	10

Sequencing and biosynthetic gene cluster prediction. Sub-cultured WAC-288 strains were grown in tryptic soy broth media for 2 days. gDNA was isolated using a method adapted from Nikodinovic *et al.* (without achromopeptidase)[4]. gDNA was sequenced using Pacific Biosciences (PacBio) RS II Sequencing Technology (Genome Quebec)[5]. Biosynthetic gene clusters within the consensus sequence were predicted using antiSMASH[6]. Complete annotated genome was deposited in Genbank CP027022.1.

Phylogenetic tree. A multilocus phylogeny was constructed using a contiguous sequence of six housekeeping genes (16S rRNA, *rpoB, atpD, gyrB, recA* and *trpB*) to phylogenetically identify WAC-288 in relation to other *Streptomyces* species[7]. The phylogeny was inferred using the Maximum Likelihood method and Tamura-Nei model[8]. Bootstrap consensus was inferred from 50 replicates[9]. The percentage of replicate trees in which the associated taxa clustered together on the bootstrap test are shown next to the branches. Evolutionary analysis was conducted in Mega X[10].

Inactivation of cosmomycin-D and 2-methylisoborneol biosynthesis. Three genes of three cosmomycin-D biosynthetic genes (cosD -*orf1219*, -*orf1222*, -*orf1245*) and one biosynthetic gene within the 2-methylisoborneol cluster (*2-mib-919*) were selected for disruption. They were individually introduced into separate pOJ260 plasmids which has an Apr^R cassette[3, 11]. Plasmids were transformed into *E. coli* ET12567 and conjugated into WAC-288. Apr^R strains were selected and correct deletions were confirmed with PCR.

Testing outbred fruit flies and other *Drosophila* species. Twelve stocks of *D. melanogaster* from the *Drosophila* Genetic Reference Panel (DGRP)[12] were crossed in 6 pairs (See table below). Those crossed fly stocks along with *D. virilis, D. pseudoobscura, D. yakuba, D. suzukii,* and *D. simulans* were tested in spore susceptibility and culture preference assays in the same manner as previously described with domesticated Canton-S *D. melanogaster* stocks.

Cross	Female		Male
**1**	DGRP-843	x	DGRP-417
**2**	DGRP-291	x	DGRP-517
**3**	DGRP-57	x	DGRP-399
**4**	DGRP-799	x	DGRP-208
**5**	DGRP-345	x	DGRP-491
**6**	DGRP-357	x	DGRP-195

Supplementary References

1. Kronheim, S., *et al.* A chemical defence against phage infection. *Nature*, **564**(7735), 283. (2018).

2. Skinnider, M. A., Merwin, N. J., Johnston, C. W., & Magarvey, N. A. PRISM 3: expanded prediction of natural product chemical structures from microbial genomes. *Nucleic acids research*, **45**(W1), W49-W54. (2017).

3. Bierman, M. et al. Plasmid cloning vectors for the conjugal transfer of DNA from *Escherichia coli to Streptomyces* spp. *Gene*, **166**, 43-49 (1992)

4. Nikodinovic, J., Barrow, K. D., & Chuck, J. A. High yield preparation of genomic DNA from Streptomyces. *Biotechniques*, **35**(5), 932-936. (2003).

5. Rhoads, A., & Au, K. F. PacBio sequencing and its applications. *Genomics, proteomics & bioinformatics*, **13**(5), 278-289. (2015).

6. Blin, K., Shaw, S., Steinke, K., et al. antiSMASH 5.0: updates to the secondary metabolite genome mining pipeline. *Nucleic acids research*, **47**(W1), W81-W87. (2019).

7. Guo, Y., Zheng, W., Rong, X., & Huang, Y. A multilocus phylogeny of the Streptomyces griseus 16S rRNA gene clade: use of multilocus sequence analysis for streptomycete systematics. International journal of systematic and evolutionary microbiology, **58**(1), 149-159. (2008).

8. Tamura, K. and Nei M. Estimation of the number of nucleotide substitutions in the control region of mitochondrial DNA in humans and chimpanzees. *Molecular Biology and Evolution* **10**:512-526. (1993).

9. Felsenstein J. Confidence limits on phylogenies: An approach using the bootstrap. *Evolution* **39**:783-791. (1985).

10. Kumar S., Stecher G., Li M., Knyaz C., and Tamura K. MEGA X: Molecular Evolutionary Genetics Analysis across computing platforms. *Molecular Biology and Evolution* **35**:1547-1549. (2018).

11. Thomas, M. G., Chan, Y. A. and Ozanick S. G. Deciphering Tuberactinomycin Biosynthesis: Isolation, Sequencing, and Annotation of the Viomycin Biosynthetic Gene Cluster. *Antimicrob. Agents Chemother.* **47**(9), 2823-2830 (2003)

12. Mackay, T. F., et al. The Drosophila melanogaster genetic reference panel. *Nature*, **482**(7384), 173-178. (2012).
