# Supplementary material for: Chemical entrapment and killing of insects by bacteria
Source: Nat Commun. 2020 Sep 14;11:4608. doi: 10.1038/s41467-020-18462-0 (PMC7490686; doi:10.1038/s41467-020-18462-0)
Supplement: Supplementary file 3 — Description of Additional Supplementary Files [file 41467_2020_18462_MOESM3_ESM.pdf]

Description of Additional Supplementary Files

File Name: Supplementary Movie 1
Description: Fruit flies are repelled by high concentrations of pure 2-MIB. Three hour video timelapse showing ~50 adult flies subject to a Y-maze choice assay. Flies were given a choice to move towards a tube containing a Whatman paper absorbed with 100 µL of mineral oil, or 5 mg of 2-MIB dissolved in the same amount of mineral oil.
