# Supplementary material for: Chemical entrapment and killing of insects by bacteria
Source: Nat Commun. 2020 Sep 14;11:4608. doi: 10.1038/s41467-020-18462-0 (PMC7490686; doi:10.1038/s41467-020-18462-0)
Supplement: Supplementary file 5 — Reporting summary [file 41467_2020_18462_MOESM5_ESM.pdf]

nature research

Corresponding author(s): Justin Nodwell
Last updated by author(s): Aug 14, 2020

Reporting Summary

Nature Research wishes to improve the reproducibility of the work that we publish. This form provides structure for consistency and transparency in reporting. For further information on Nature Research policies, see our Editorial Policies and the Editorial Policy Checklist.

Statistics

For all statistical analyses, confirm that the following items are present in the figure legend, table legend, main text, or Methods section.

n/a	Confirmed	
☐	☒	The exact sample size (*n*) for each experimental group/condition, given as a discrete number and unit of measurement
☐	☒	A statement on whether measurements were taken from distinct samples or whether the same sample was measured repeatedly
☐	☒	The statistical test(s) used AND whether they are one- or two-sided<br>*Only common tests should be described solely by name; describe more complex techniques in the Methods section.*
☒	☐	A description of all covariates tested
☒	☐	A description of any assumptions or corrections, such as tests of normality and adjustment for multiple comparisons
☐	☒	A full description of the statistical parameters including central tendency (e.g. means) or other basic estimates (e.g. regression coefficient) AND variation (e.g. standard deviation) or associated estimates of uncertainty (e.g. confidence intervals)
☒	☐	For null hypothesis testing, the test statistic (e.g. *F*, *t*, *r*) with confidence intervals, effect sizes, degrees of freedom and *P* value noted<br>*Give P values as exact values whenever suitable.*
☒	☐	For Bayesian analysis, information on the choice of priors and Markov chain Monte Carlo settings
☒	☐	For hierarchical and complex designs, identification of the appropriate level for tests and full reporting of outcomes
☒	☐	Estimates of effect sizes (e.g. Cohen's *d*, Pearson's *r*), indicating how they were calculated

Our web collection on statistics for biologists contains articles on many of the points above.

Software and code

Policy information about availability of computer code

Data collection	No software was used for data collection.
Data analysis	antiSMASH 5.0 (Bacterial version) for genome-wide analysis of secondary metabolite biosynthetic gene clusters. https://<br>MassLynx Mass Spectrometry Software V4.1 and MZmine 2 for MS data.<br>Morpheus (Broad Institute) (https://software.broadinstitute.org/morpheus) Dec 2019 for heat map generation.<br>Fiji (ImageJ 1.52p) for fluorescence microscopy image processing.<br>Microsoft Excel for Office 365 MSO (16.0.12527.20880) 64-bit was used for two-tailed t-test and P-value calculations<br>Graphpad PRISM 6 for dot-plot graph generation

For manuscripts utilizing custom algorithms or software that are central to the research but not yet described in published literature, software must be made available to editors and reviewers. We strongly encourage code deposition in a community repository (e.g. GitHub). See the Nature Research guidelines for submitting code & software for further information.

Data

Policy information about availability of data

All manuscripts must include a data availability statement. This statement should provide the following information, where applicable:

- Accession codes, unique identifiers, or web links for publicly available datasets
- A list of figures that have associated raw data
- A description of any restrictions on data availability

The genome sequence that supports this study has been deposited in GenBank with the accession code CP027022.1 [https://www.ncbi.nlm.nih.gov/nuccore/CP027022.1]. The authors declare that all other data supporting the findings of this study are available within the paper and its supplementary files.

Field-specific reporting

Please select the one below that is the best fit for your research. If you are not sure, read the appropriate sections before making your selection.

- [x] Life sciences
- [ ] Behavioural & social sciences
- [ ] Ecological, evolutionary & environmental sciences

For a reference copy of the document with all sections, see nature.com/documents/nr-reporting-summary-flat.pdf

Life sciences study design

All studies must disclose on these points even when the disclosure is negative.

Sample size	For behavioral experiments involving adult fly preference and larval development, no sample size calculation was carried out. Sample sizes were chosen based on the maximum number of organisms that could be placed in the assay setup without overcrowding. Sample sizes were deemed appropriate once the results were reproducible and significantly different between conditions.
Data exclusions	No data was excluded in this study.
Replication	Larval development assays +extract/spores were repeated individually in triplicate. Replicates of adult behavioral preference were repeated 6 - 13 times as indicated in the methods.
Randomization	Allocation of organisms into assay conditions were randomized. All Drosophila melanogaster stocks were maintained under consistent conditions as described until they were allocated for sample testing. This includes larvae that were synchronized for embryo hatching prior to sample testing (i.e. laid by adult flies within a 5 hr time-window prior to 24 hr incubation). Adult flies used for preference assays were randomly allocated for behavioural assays within stocks that were 2-3 weeks old. Flies were transferred to assays from the same stock were divided into equal groups.
Blinding	Blinding was not carried out in the design of this study. We acknowledge the limitations and potential biases introduced by the lack of blinding however efforts to maintain consistency between fly populations were carried out as described in the Methods.

Reporting for specific materials, systems and methods

We require information from authors about some types of materials, experimental systems and methods used in many studies. Here, indicate whether each material, system or method listed is relevant to your study. If you are not sure if a list item applies to your research, read the appropriate section before selecting a response.

Materials & experimental systems

n/a	Involved in the study	
☐	☒	Antibodies
☐	☒	Eukaryotic cell lines
☒	☐	Palaeontology and archaeology
☐	☒	Animals and other organisms
☒	☐	Human research participants
☒	☐	Clinical data
☒	☐	Dual use research of concern

Methods

n/a	Involved in the study	
☒	☐	ChIP-seq
☒	☐	Flow cytometry
☒	☐	MRI-based neuroimaging

Antibodies

Antibodies used	Cleaved Caspase-3 (Asp175) Antibody (Cell Signaling Technology #9661) (Rabbit polyclonal) Lot #45<br>Cy3 AffiniPure Donkey Anti-Mouse IgG (Jackson ImmunoResearch) Lot #147730
Validation	http://media.cellsignal.com/pdf/9661.pdf<br>https://www.jacksonimmuno.com/catalog/products/715-165-150

Eukaryotic cell lines

Policy information about cell lines

Cell line source(s)	S. cerevisiae (BY4741; MATa his3Δ1 leu2Δ0 met15Δ0 ura3Δ0) (Brenda Andrews), C. albicans (CaLC155) (Leah Cowen)
Authentication	No authentication was used.
Mycoplasma contamination	Cell lines were not tested for mycoplasma contamination.

Commonly misidentified lines (See ICLAC register)	No commonly misidentified lines were used.

Animals and other organisms

Policy information about studies involving animals; ARRIVE guidelines recommended for reporting animal research

Laboratory animals	Drosophila melanogaster (Canton-S), D. virilis, D. suzukii, D. yakuba, D. simulans and D. pseudoobscura.<br>Larvae synchronized hatched from embryo between 24 - 30 hrs old.<br>Adults tested between (3 - 4 weeks old / 1 - 2 weeks old after eclosion)<br>Gender/Sex randomized.
Wild animals	No wild animals were used in this study.
Field-collected samples	Information regarding the Wright Actinomycete Collection which are field-collected samples can be found here: http://www.thewrightlab.com/wright-actinomycete-collection
Ethics oversight	No ethical oversight was required for work with Drosophila.

Note that full information on the approval of the study protocol must also be provided in the manuscript.
