# Supplementary material for: Chemical entrapment and killing of insects by bacteria
Source: Nat Commun. 2020 Sep 14;11:4608. doi: 10.1038/s41467-020-18462-0 (PMC7490686; doi:10.1038/s41467-020-18462-0)
Supplement: Supplementary file 2 — Peer Review File [file 41467_2020_18462_MOESM2_ESM.pdf]

Editorial Note: This manuscript has been previously reviewed at another journal that is not operating a transparent peer review scheme. This document only contains reviewer comments and rebuttal letters for versions considered at *Nature Communications*.

REVIEWERS' COMMENTS:

Reviewer #2 (Remarks to the Author):

The manuscript has been improved and my concerns were discussed or corrected. I now think the article is fien to publish. No objections.

Reviewer #3 (Remarks to the Author):

The author did a good job revising their manuscript.
